# Goal-Directed Mobility of Medical Inpatients–A Mini Review of the Literature

**DOI:** 10.3389/fmed.2022.878031

**Published:** 2022-05-18

**Authors:** Jeannelle Heinzmann, Christine Baumgartner, Fabian D. Liechti

**Affiliations:** Department of General Internal Medicine, Inselspital, Bern University Hospital, University of Bern, Bern, Switzerland

**Keywords:** physiotherapy, goal-attainment, goal-directed mobilization, mobility, internal medicine, hospital medicine

## Abstract

**Background:**

Inpatients spend most of their hospitalization in bed, which can lead to negative physical, social, and psychological outcomes, especially in the geriatric population. Goal-directed mobilization involves setting mobility goals with patients and care teams working together toward achieving these goals.

**Methods:**

Three different platforms (SCOPUS, Ovid Medline, PubMed) were searched. Search terms included “goal-directed,” “goal-attainment” or “goal-setting,” and “inpatient” or “hospitalization” and “mobility” or “mobilization.” Articles were included if mobility goals were set in acutely hospitalized adults. Studies were excluded if only covering specific illness or surgery.

**Results:**

One Hundred Seventy three articles were screened for inclusion by two independent reviewers. In the final analysis, 13 articles (5 randomized controlled trials, 2 *Post-hoc* analyses, 3 quality-improvement projects, 1 pre-post two group analysis, 1 comment and 1 study protocol) were assessed. Goal-directed mobilization improved mobility-related outcomes, i.e., level of mobilization, activity, daily walking time and functional independence. Readmissions, quality of life, discharge disposition and muscle weakness were not significantly altered and there was conflicting evidence regarding length of stay and activities of daily living.

**Conclusion:**

There is a lack of evidence of goal-directed mobilization on relevant outcomes due to the low number of studies in the field and the study design used. Further research on goal-directed mobility should use standardized mobility protocols and measurements to assess mobility and the effects of goal-directed mobility more accurately and include broader patient populations.

## Introduction

Acutely hospitalized medical patients spend up to 83% of the time in bed (1, 2). Furthermore, sedentary time increases throughout hospitalization (3). Although bedrest is nowadays prescribed infrequently during hospitalization (4), the notion in patients and providers remains that bedrest is a therapeutic necessity (5). Low ambulation in the last 24 h of hospital stay and a decline in mobility during hospitalization were associated with an increased risk of death after discharge (6).

Especially elderly patients experience adverse functional changes during their hospital stay, such as reduction in Activities of Daily Living (ADL) function (7), with only about 30% able to regain their level of self-care ADL after 1 year of discharge (8). Additionally, low mobility can lead to new institutionalization (5), pressure ulcers (9) and reduced aerobic capacity (10). However, immobility is irrespective of patient age, implying that barriers to mobility are not limited to the elderly (11).

Increasing patients' mobility during hospitalization results in shorter length of stay, improved aerobic capacity and reduction of pulmonary embolism (12), and reduced risk of decline in ADL ability, nursing home residence and 1-month mortality (13).

Early mobilization has been implemented as standard of care in many surgical disciplines (14) and begun to find its way in intensive care units (ICU) (15, 16). However, there seems to be little evidence of implementation, effects, and outcomes of mobilization in hospitalized general medical patients.

The aim of this review was to investigate evidence on goal-directed mobilization (GDM) of adult medical inpatients, where patients or care teams set well-defined mobility goals for improving mobility during hospitalization.

## Methods

### Study Design

This study is a comprehensive narrative review, using the Preferred Reporting Items for Systematic reviews and Meta-Analyses (PRISMA) statement for reporting the results (17).

### Search Strategy

We searched the following databases on 12.07.2021: Ovid Medline, PubMed, and Scopus. The search strategy included the terms “goal-directed,” “goal-attainment” or “goal-setting,” and “inpatient” or “hospitalization” and “mobility” or “mobilization.” The full search is depicted in the Supplementary Material. Additionally, we screened the bibliographies of included articles, editorials, and guidelines on mobility interventions.

### Selection Criteria

Studies were included if they reported on interventions or investigations targeting goal-directed mobilization in hospitalized internal medicine patients. Although most articles on the topic focus on geriatric patients, we included all articles including adult patients to be more inclusive. Articles were included irrespective of the study design or language.

Articles were excluded if they did not specifically outline mobility or mobilization or if they focused on surgery or specific illnesses, e.g., stroke, multiple sclerosis, spinal cord injury, hip fracture, or coronary heart disease.

### Study Procedure

Two independent reviewers (JH, FDL) examined the search results regarding inclusion and exclusion criteria based on title and abstract. Disagreements were solved with discussion until consensus was reached. Included papers were read in a systematic approach, focusing on type of study, study goals, the setting, methods, measurements, and the results and conclusion. The study results were qualitatively synthesized and narratively described.

## Results

**Figure 1:**
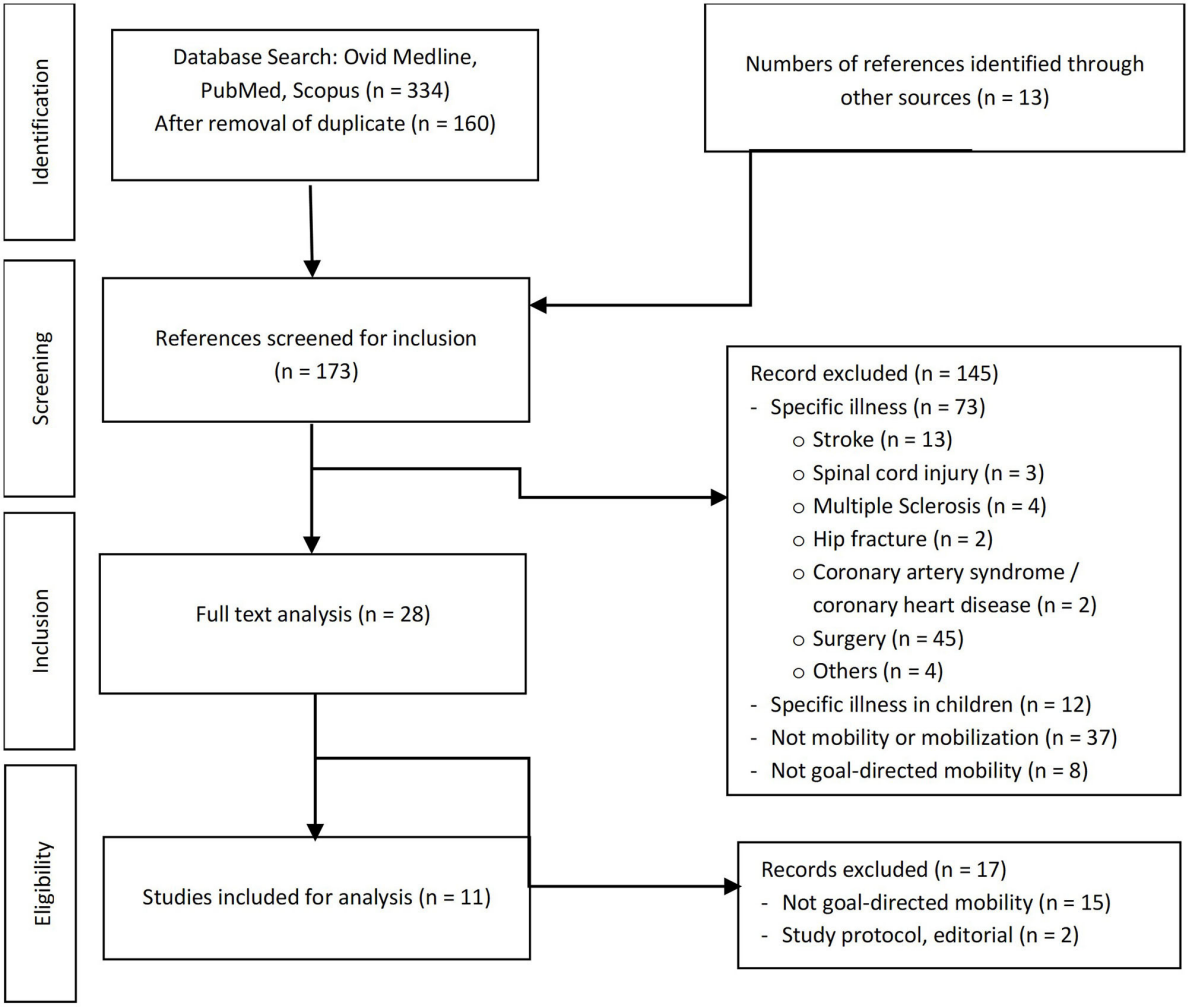
Flow chart of search process.

The search resulted in 334 articles (Figure 1). After removing 174 duplicates, 160 articles remained in total. As 145 articles did not meet the eligibility criteria, they were excluded. Of these 145 papers, 73 were excluded, as they focused on specific illnesses, such as surgery (45), stroke (13), spinal cord injury (3), multiple sclerosis (4), hip fracture (2), coronary heart or coronary artery disease (2) and 4 others. Twelve articles focused on specific illnesses or rehabilitation in children and were excluded. Furthermore, 37 articles were removed, because they did not investigate mobility or mobilization. An additional 8 articles were removed as mobility was not investigated in a goal-direct, goal-attainment or goal-setting manner. Additionally, 15 articles were excluded, as the investigations or interventions were conducted out of hospital.

As such, 15 articles were read in full text and references were screened for additional sources. An additional 13 papers were added to full text analysis. As 15 papers did not investigate goal-directed mobility or mobilization, they were excluded and therefore, 13 articles were included for the analysis (Table 1). Of the 13 remaining papers screened for full text, five were randomized controlled trials (RCT) (16, 18–21), two were *post hoc* analyses of one of the RCTs (22, 23), three were “quality improvement projects” (24–26), and one was a pre-post two group analysis (27). A protocol for a randomized controlled trial (15) and a comment on an aforementioned RCT (28) were excluded from final analysis.

**Table 1:** Overview of studies included in the final analysis.

**References**	**Type of study**	**Setting, population**	**Interventions**	**Main findings**
Brown et al. (18)	RCT	−100 patients on medical wards	- Intervention: assisted ambulation and behavioral intervention with mobility goals - Control: usual care	- Significantly higher LSA at 1-month post-hospitalization in intervention vs. control group - No significant change in ADL
Exum and Hull (25)	Quality improvement pilot study	- Mobility group: 292 patients (general medicine and general surgical ward)	- Intervention: JH-HLM scale for mobilizing patients and setting daily goals with the help of a mobility technician	- Changes in JH-HLM score, falls, length of stay, discharges to home were not significant after the intervention - Intervention could be cost-effective
Hodgson et al. (19)	RCT	−50 ventilated patients in ICU	- Intervention: active functional activities led by physical therapy mobility team - Control: unit practice	- Higher levels of activity in intervention vs. control group - Greater duration of exercise
Hoyer et al. (26)	Quality improvement project	−3,352 patients on 2 general medicine units	- Mobilizing patients, JH-HLM for setting daily goals	- Shorter length of stay after intervention - Percentage of patients with JH-HLM ≥ 6 increased from 43 to 70% and improvement in mobility increased from 32 to 45%
Peel et al. (20)	RCT	−270 patients on Geriatric Rehabilitation Units	- Intervention: accelerometer data for setting mobility goals - Control: usual care	- Significantly higher non-therapy walking time (by 7 min per day) in intervention vs. control group - Median daily walking time increased from 10.3 to 32.1 min (IG) vs. 9.5 to 26.5 min (CG) at day 28
Schaller et al. (16)	RCT	−200 ventilated patients in the surgical ICU	- Intervention: facilitator and team creating daily goal, posted at patients' bedside - Control: practice guidelines	- Higher mean achieved SOMS level in intervention vs. control group - Higher level of mobility at discharge - Shorter length of stay - Higher functional independence
Teodoro et al. (21)	RCT	−48 medical-surgical patients	- Intervention: education, daily goals, reminders - Control: usual care	- Ambulation significantly improved on the 3rd day
Klein et al. (24)	Qualitative improvement project	- Two adult care units - 1,966 patients at baseline vs. 2,164 patients on project unit	- Intervention: JH-HLM goal displayed in patients' room - Control: control unit	- Meeting and exceeding mobility scores more often and higher levels of mobility
Cohen et al. (27)	Quasi-experimental pre-post two group analysis	- Two internal medicine units - 377 patients	- Intervention: education, walking 900 steps daily, removing mobility obstacles	-Intervention group walked significantly more - Significantly lower odds of decline in basic ADL and community mobility
Schaller et al. (22)	*Post-hoc* analysis of RCT	- *Post-hoc* analysis of SOMS trial	- Influence of the initial level of consciousness on early GDM	- Early GDM effective in increasing likelihood of patient with initial impairment of consciousness to leave hospital functionally independent
Scheffenbichler et al. (23)	*Post-hoc* analysis of RCT	- *Post-hoc* analysis of SOMS trial	- Effect of acuity of illness on early GDM	- Speed of mobility recovery was significantly higher in patients with moderate acuity - Patients with low acuity are in less need of early GDM

*RCT, Randomized Controlled Trial; LSA, Life-Space Assessment; ADL, Activities of Daily Living, JH-HLM, John Hopkins Highest Level of Mobility; ICU, Intensive Care Unit; IG, intervention group; CG, control group; SOMS, SICU optimal mobilization score; GDM, goal-directed mobilization*.

In intervention studies, the comparator was usually “standard of care”, not involving mobility goals, except for one study (20).

### Early Goal-Directed Mobilization in the ICU

One study involved 200 mechanically ventilated patients (mean age 65 years) on a surgical ICU (16). A daily goal based on the surgical ICU optimal mobilization score (ranging from 0 = no mobilization to 4 = ambulation) was defined by a facilitator and the clinical team and a sign with the target goal was posted at patients' bedside. Early GDM led to higher levels of mobilization measured by this score at ICU discharge, significantly shorter length of ICU stay (7 vs. 10 days), and higher mobility related functional independence at hospital discharge. No effect 3 months after hospital discharge was found regarding muscle weakness and quality of life. However, in both intervention and control group more than half of the participants were lost to follow-up. Further limitations include no blinding for bedside clinicians, lack of generalizability, and different protocols for the control group across study centers (16).

Another early GDM protocol in the ICU involving 50 invasively ventilated patients incorporated active functional activities, such as walking, standing, sitting, and rolling. Patients' goal was to do active exercise with a mobility team (19). The ICU mobility scale was used to calculate the amount of time spent doing active exercise (ranging from 30 to 60 min). Patients in the intervention group experienced higher levels of activity measured by this scale and had a greater duration of active exercise each day (20 min/d in early GDM vs. 7 min/d in the control group). At follow-up 6 months after randomization, there were no differences regarding health-related quality of life, anxiety and depression, ADL, and return to work. However, there was a lower average time of active exercise recorded than prescribed in the study protocol. Furthermore, the sample size was insufficient to detect clinically relevant differences in outcomes. Additionally, participants in the intervention group (mean age: 64 years) were older than the control group (mean age: 53 years) (19).

A *post-hoc* analysis showed that in both groups of patients with low and high Glasgow Coma Scale, early GDM led to a significant increase in functional independence at hospital discharge (22). Patients with impaired level of consciousness could be safely and effectively mobilized (22).

Another *post-hoc* analysis focusing on the acuity of patients' illness concluded that speed of mobility recovery was significantly higher in patients with illness of moderate acuity receiving early GDM (23). Furthermore, patients with moderate acuity illness in the control group had the lowest probability of reaching functional independence. As such, there is an unrecognized need for mobilization therapy in this group (23).

Both *post-hoc* analyses faced similar limitations, as they were designed as *post-hoc* analyses of a RCT trial (16), where interaction and subgroup analyses were not originally planned (22). Generalisability of the results to all critically ill patients may not be possible (22, 23).

### Accelerometry in Geriatrics

Accelerometers monitored patient's activity in a RCT with 255 patients from a geriatric unit (mean age: 81 years) (20). The intervention group and their therapists were informed about the participants' walking time using daily accelerometer data. Therapists and patients set mobility goals together, including targets for daily walking time. The objectives were re-evaluated weekly and modified according to accelerometer data. In-therapy and non-therapy walking time over 28 days were compared. Non-therapy walking time in the intervention group was higher by about 7 min/day. The median daily walking time, consisting of non-therapy and in-therapy walking time, increased from 10.3 to 32.1 min per day in the intervention group, and from 9.5 to 26.5 min per day in the control group. Median length of stay, readmissions, discharges to a higher level of care, improvements in the short physical performance battery, health related quality of life, and the ADL scale were not significantly different. Although different models of accelerometers were used, the proportions of patients using each device was not significantly different (20).

### Acute Care Setting

In a single-blinded RCT, the mobility program consisted of assisted ambulation twice daily and daily mobility goals for additional activity (18). The study included 100 patients with a mean age of 74 years in a veterans study (97% male). Functional outcome was assessed by ADL, measured at admission, retrospectively for 2 weeks prior to admission, at discharge and at 1-month follow-up. Community mobility was assessed by the Life-Space Assessment (LSA), which measures mobility based on the distance reported moving during the last 4 weeks. The score ranges from 0 to 120, with higher scores representing greater mobility. LSA was assessed at baseline and 1-month follow-up. ADL did not significantly change between the groups. However, significant difference in LSA was measured, as the intervention group had a 10-point higher LSA score at 1-month posthospitalization (18).

The STEP-UP program in an inpatient medical-surgical unit investigated 48 patients over a 3-day period (21). The intervention consisted of education, setting daily ambulation goals, and giving ambulation reminders. Patient ambulation was assessed using a pedometer. The amount of ambulation significantly increased on the third day for the intervention group, but not before (21).

Klein et al. designed a quality-improvement project comparing two adult care units (*n* = 2,164 patients) with each other and to baseline (1,966 patients) (24). Patients had a mean age of 52 years. Patients' goal was a mobility level based on the John Hopkins Highest Level of Mobility (JH-HLM) scale (range: 1 = “lying in bed” to 8 = “walking 250 feet or more”). The goal was posted at patient's bedside, and nursing staff was instructed to implement the intervention. Patients on the project unit met their daily mobility goals more frequently and had higher levels of mobility. JH-HLM scores were significantly higher in the project unit, but no difference in mean JH-HLM goal was found between intervention and control group. The project unit had a higher probability of reaching maximum JH-HLM scores of 3 or greater, and a more than 70% probability of reaching an ambulatory status (JH-HLM ≥ 6) during hospital stay (compared to 60% in the control unit) (24). It was not investigated whether the intervention had effects on length of stay, pressure injuries, number of falls, or number of discharges to rehabilitation facilities. Further limitations include lack of randomization and blinding. Additionally, the project unit included more stroke patients than the control unit (24).

A quality-improvement project on two general medicine units involved 3,352 patients with a mean age of 54 years. Pre-existing staff mobilized patients 3 times/day and daily mobility goals were set. The JH-HLM was used to assess mobility and create daily goals. It was concluded that this intervention was safe and cost-effective, as there was a reduced mean length of stay, but no difference in injurious falls. Patients reaching the highest ambulatory level (JH-HLM ≥ 6) increased from 43 to 70% and improvement in mobility increased from 32 to 45%. Due to the study design, no direct cause-and-effect relationship can be drawn between the intervention and improvement of mobility and reduction of length of stay. Furthermore, there was a higher rate of missing documentation in the first 4 months (26).

Another quality improvement mobility program investigated 954 patients on a surgical and general medical unit at baseline and project period. The mobility program group consisted of 292 patients with a mean age of 64 years on the general medicine ward and 59 years on the general surgical ward. A mobility technician assessed patient's JH-HLM score and mobilized the patient with the goal of increasing the score a minimum of one level per day. Furthermore, the patient was given a goal to improve the score one level daily. Although the JH-HLM score did not change significantly, the goal of improving patients' mobility by one level was achieved. Falls and length of stay decreased in medical patients, and they were more frequently discharged home instead of transferred to rehabilitation facilities. However, none of these outcomes reached statistical significance. Yet, definite conclusions cannot be drawn due to small sample size, short pilot duration, and limited data on demographics of the baseline data group (25).

The WALK-FOR program used a pre-post two-group comparative design to prevent functional decline in older adults (27). It was set in two internal medicine units with 377 patients, 65 years (mean age 75 years) or older. The intervention consisted of addressing the main obstacles to mobility, education, setting the goal of walking 900 steps per day, and removing all mobility obstacles. Nurses assessed participants and steps were counted by an accelerometer. Patients in the intervention group walked significantly more, and the odds of reduction in basic ADL function and community mobility was significantly reduced. There was no significant effect on prevention of decline in instrumental ADL (27).

## Discussion

This review examined publications involving GDM in hospitalized patients and identified 11 papers investigating multiple interventions in different settings, using various outcomes, which are challenging to compare (16, 18–27). Only seven studies (18, 20, 21, 24–27) investigated its effect in patients outside of the ICU setting in general medicine.

While mobility is a relevant topic, there seems to be a lack of standard, how interventions should be conducted, assessed, and measured. The quality of evidence is generally low due to the observational study design used in most studies. We found only five RCTs (16, 18–21), all of which could not be blinded; however, outcome assessment was blinded in four trials (16, 18–20). There were three quality improvement projects (24–26), where direct cause-and-effect relationships are difficult to be identified.

Setting mobility goals led to higher levels of mobilization, activity and mobility-related functional independence, a higher probability of reaching ambulatory status, and increased daily walking time and community mobility (Table 2). Quality of life, readmissions, discharge dispositions, and muscle weakness were not significantly affected by setting mobility goals. The reasons why mostly secondary outcomes were not significantly different could be due to small sample sizes, lack of power, significant loss of follow-up or the study design being used. Further research is needed to draw definite conclusions. The effect of GDM on ADL function was ambiguous; three studies found no difference in ADL function (18–20), while one study in elderly patients saw a significant reduction of decline in basic ADL, but no significant effect on decline in instrumental ADL (27).

**Table 2:** Overview of effects evaluated in the included studies.

**Item**	**Effect**	**Reference**
Mobilization/Mobility	Significantly higher levels	(16, 24, 26)
Activity	Significantly higher levels	(19)
Mobility-related functional independence	Significantly higher levels	(16)
Ambulatory status	Significantly improved Higher probability of reaching ambulatory status	(21, 24, 26)
Daily walking time	Significantly increased	(20)
Community mobility	Significantly higher 1 month post hospitalization Significantly lower odds of decline	(18, 27)
Quality of life	No significant changes	(16, 19, 20)
Readmissions	No significant changes	(20)
Discharge dispositions	No significant changes	(20, 25)
Muscle weakness	No significant changes	(16)
Activity of Daily Living	No significant changes Significantly lower odds of decline in basic Activities of Daily Living	(18–20) (27)
Length of stay	No significant changes Significant reduction	(19, 20, 25) (16, 26)
Long-term outcomes	No significant changes Significant improvements in community mobility 1 month post hospitalization	(16, 19, 26) (18)

GDM seemed to improve short-term outcomes, such as walking time or levels of mobilization at hospital discharge. Long-term outcomes, assessed between 28 days and 6 months, were only investigated in four papers (16, 18–20) and were not significantly altered by GDM, except for one, where community mobility was significantly improved at 1-month follow-up (18).

Economic factors were investigated in one study, concluding that a mobility technician could be cost-effective by helping reduce length of stay and discharge to post-acute rehabilitation facilities. However, these outcomes were not significantly altered (25). Two trials created new roles for implementing the intervention (16, 25), which could increase costs, while the others used pre-existing staff (18–20, 24, 26, 27). If GDM can reduce length of stay, this could be a cost-effective intervention. Three studies concluded that length of stay was not significantly different (19, 20, 25), while two study reported reduced length of surgical ICU or hospital stay when implementing GDM (16, 26). Further research is needed to investigate, whether GDM can shorten length of stay as important economic parameter.

In rehabilitation, goal attainment scaling seems to be a valid, reliable, and sensitive outcome measure for patients according to a systematic review from 2006 (29). In stroke rehabilitation and rehabilitation after ICU discharge, goal setting led to higher patient satisfaction when meeting their goals (30, 31). Adjusting the goals to patients' changing needs and improvements may be the reason, why mobility and activity levels increased in GDM intervention groups. Setting and achieving mobility goals could improve patients' motivation and thus encourage patients to stay more active during hospitalization.

It has been established earlier that a standardized mobility protocol can lead to higher functional status and reduced length of stay in different patient groups (32). As such, mobility programs such as the Mobilization of Vulnerable Elders in Ontario (MOVE ON) have been created, where increased mobilization and reduced length of stay were observed (33, 34).

A systematic review investigating early mobilization in the ICU concluded that early mobilization is feasible, safe and leads to a greater achievement of mobility milestones (35). Nevertheless, questions remain concerning the effects of non-mobility related outcomes, such as muscle weakness, quality of life and length of stay. As such, there is need for more research to answer these questions.

There are some limitations to this review. First, only a small number of papers could be included. However, there seems to exist little evidence on this specific topic and using three relevant databases we are confident to have included the relevant literature. Second, although we did not systematically appraise the quality and strength of evidence, we used a systematic approach to analyze the relevant literature, focusing on type of study, study goals, the setting, methods, measurements, and the results and conclusion. Third, only articles in English were investigated in this review. Yet, articles in different languages were not excluded, there simply were not any articles that fit the criteria. Fourth, it can be assumed that other relevant biases such as publication bias and effects of small sample size may have influenced the findings.

Future research should target general medical inpatients, as there is little evidence of the effects of GDM in this patient group.

## Conclusion

The quality of evidence on GDM in acutely hospitalized patients is generally low due to the difficulty of blinding and popular study design (implementation studies). Setting mobility goals led to improvements in mobility-related outcomes, such as higher levels of mobilization, activity, and mobility-related functional independence, and increased daily walking time and community mobility. Quality of life, readmissions, discharge dispositions, and muscle weakness were not significantly altered. There is conflicting evidence regarding the effect of GDM on length of stay and ADL function.

Future research evaluating the effects of GDM should use more standardized programs and measurements for assessing mobility to meaningfully compare outcomes and provide evidence on how to mobilize inpatients.

## Author Contributions

CB and FDL contributed to conception and design of the study and performed the literature search and review. JH wrote the first draft of the manuscript. All authors contributed to manuscript revision, read, and approved the submitted version.

## Funding

This work was supported by Swiss Society of General Internal Medicine (SGAIM) Foundation, Bern, Switzerland, and Foundation Sana (grant number GF 2021-0048), Bern, Switzerland. Open access funding was provided by the University of Bern.

## Conflict of Interest

The authors declare that the research was conducted in the absence of any commercial or financial relationships that could be construed as a potential conflict of interest.

## Publisher's Note

All claims expressed in this article are solely those of the authors and do not necessarily represent those of their affiliated organizations, or those of the publisher, the editors and the reviewers. Any product that may be evaluated in this article, or claim that may be made by its manufacturer, is not guaranteed or endorsed by the publisher.

## Abbreviations

ADL: Activities of Daily Living
GDM: Goal-directed Mobility
ICU: Intensive Care Unit
JH-HLM: John Hopkins Highest Level of Mobility
LSA: Life-Space Assessment
RCT: Randomized Controlled Trial.
