# Erratum: Goal-directed mobility of medical inpatients—A mini review of the literature

**DOI:** 10.3389/fmed.2023.1183717

**Published:** 2023-03-22

**Authors:** Frontiers Production Office

**Affiliations:** Frontiers Media SA, Lausanne, Switzerland

**Keywords:** physiotherapy, goal-attainment, goal-directed mobilization, mobility, internal medicine, hospital medicine

An omission to the funding section of the original article was made in error. The following sentence has been added: “Open access funding was provided by the University of Bern.”

The original article has been updated.

