# Supplementary material for: Goal-Directed Mobility of Medical Inpatients–A Mini Review of the Literature
Source: Front Med (Lausanne). 2022 May 18;9:878031. doi: 10.3389/fmed.2022.878031 (PMC9158316; doi:10.3389/fmed.2022.878031)
Supplement: Supplementary file 1 [file Data_Sheet_1.docx]

# Supplementary material

**Full search**

Scopus 12.07.2021

(TITLE-ABS-KEY ("goal-directed" OR "goal directed" OR "goal attainment" OR "goal-attainment" OR "goal setting" OR "goal-setting")

TITLE-ABS-KEY (mobil* OR mobili?ation)

TITLE-ABS-KEY (inpatient* OR hospit*)

1 and 2 and 3

PubMed 12.07.2021

inpatient*[Title/Abstract] OR inpatient[Title/Abstract] OR hospital*[Title/Abstract] OR hospital[Title/Abstract] OR hospitalisation[Title/Abstract] OR hospitalization[Title/Abstract]) OR (hospitalised[Title/Abstract])) OR (hospitalized[Title/Abstract]

mobilisation[Title/Abstract] OR mobilization[Title/Abstract] OR mobil*[Title/Abstract] OR mobility[Title/Abstract]

goal-directed"[Title/Abstract] OR "goal directed"[Title/Abstract] OR "Goal-attainment"[Title/Abstract] OR "goal attainment"[Title/Abstract] OR "goal setting"[Title/Abstract] OR "goal-setting"[Title/Abstract]

1 and 2 and 3

Ovid Medline 12.07.2021

("Goal-directed" or "goal directed" or "goal-attainment" or "goal attainment" or "goal-setting" or "goal setting").ti,ab,kw.

mobili?ation.ti,ab,kw.

exp Mobility Limitation/

mobil*.ti,ab,kw.

2 or 3 or 4

exp Inpatients/

(inpatient* or hospit*).ti,ab,kw.

6 or 7

1 and 5 and 8
